# Developmental iodine deficiency and hypothyroidism impair neural development in rat hippocampus: involvement of doublecortin and NCAM-180

**DOI:** 10.1186/1471-2202-11-50

**Published:** 2010-04-23

**Authors:** Jian Gong, Wanyang Liu, Jing Dong, Yi Wang, Hongde Xu, Wei Wei, Jiapeng Zhong, Qi Xi, Jie Chen

**Affiliations:** 1 Department of Occupational and Environmental Health, School of Public Health, China Medical University, Shenyang, PR China; 2 Department of Physiology, the University of Tennessee Health Science Center, Memphis, TN 38163, USA

## Abstract

**Background:**

Developmental iodine deficiency results in inadequate thyroid hormone (TH), which damages the hippocampus. Here, we explored the roles of hippocampal doublecortin and neural cell adhesion molecule (NCAM)-180 in developmental iodine deficiency and hypothyroidism.

**Methods:**

Two developmental rat models were established with either an iodine-deficient diet, or propylthiouracil (PTU)-adulterated water (5 ppm or 15 ppm) to impair thyroid function, in pregnant rats from gestational day 6 until postnatal day (PN) 28. Silver-stained neurons and protein levels of doublecortin and NCAM-180 in several hippocampal subregions were assessed on PN14, PN21, PN28, and PN42.

**Results:**

The results show that nerve fibers in iodine-deficient and 15 ppm PTU-treated rats were injured on PN28 and PN42. Downregulation of doublecortin and upregulation of NCAM-180 were observed in iodine-deficient and 15 ppm PTU-treated rats from PN14 on. These alterations were irreversible by the restoration of serum TH concentrations on PN42.

**Conclusion:**

Developmental iodine deficiency and hypothyroidism impair the expression of doublecortin and NCAM-180, leading to nerve fiber malfunction and thus impairments in hippocampal development.

## Background

Iodine is an essential trace element that plays a vital role in the synthesis of thyroid hormones (TH). The maintenance of thyroid function directly depends on adequate availability of dietary iodine. Iodine deficiency is one of the most common, preventable causes of brain damage in the world [1], especially in China [2]. During the gestational and postnatal periods, iodine deficiency in both mother and offspring is a common cause of hypothyroidism. This is because the mother is the only source of iodine intake for the fetus and neonate during these developmental periods [3-5]. Rat pups are not able to make sufficient TH until the day of birth. Given that 3,5,3' triiodothyronine (T3) is the most functionally active form of TH, sufficient levels of both thyroxine (T4) and T3 are indispensable to mammalian brain development and metabolic homeostasis [6] by binding to nuclear thyroid hormone receptor (TR), which is a ligand-regulated transcription factor [7]. In the nucleus, the facilitated binding of T3-TR to a common nuclear receptor called RXR makes a heterodimer-T3-TR-RXR complex which in turn binds to thyroid hormone DNA response elements, regulating the consequent gene transcription through the action of co-repressors and co-activators [8]. It is well known that TRs which are expressed in the hippocampus [9] and hippocampus are highly sensitive to the actions of TH [10,11], suggesting that TH is crucial in hippocampal development.

During growth and development, structural remodeling occurs in a number of brain regions, including the hippocampus, where neural plasticity is a lifelong characteristic [12,13]. Many studies have revealed a connection between hippocampal neurogenesis and hippocampus-dependent functions [14,15]. New neurons are continuously added to the dentate gyrus (DG), allowing modulation of hippocampal function [16]. As a marker of new neurons, doublecortin is a microtubule associated protein and present in differentiating and migrating neurons [17]. Doublecortin is able to stabilize and bundle microtubules during hippocampal neurogenesis. Besides doublecortin, neural cell adhesion molecule (NCAM) is also involved in different aspects of structural plasticity [18-21]. NCAM, the first cell adhesion molecule, mediates homophilic adhesion between cells and is of crucial importance to central nervous system (CNS) development [22,23]. This protein is expressed in all neurons from very early stage during development, implying that NCAM may be an important modulator of neural plasticity in synaptic rearrangements and neuronal remodeling [24,25]. For example, NCAM-180, a specific NCAM isoform expressed in the brain [26], plays an important role in synaptic remodeling and long-term potentiation (LTP) [27]. In the new neurons, however, doublecortin and polysialylated form of NCAM act in different cellular compartments, the microtubule cytoskeleton and the plasma membrane respectively, to promote/allow migration and differentiation of immature elements [18,28,29]. Recently, a genomic analysis of subclinical hypothyroidism detected changes of doublecortin and NCAM 1 in the neocortex of the developing rat brain [30]. However, relatively little is known about whether doublecortin and NCAM-180 expressions are affected following iodine deficiency or PTU induced-hypothyroidism.

Importantly, many lines of evidence have determined that iodine deficiency-induced developmental defects of the CNS are irreversible in fetuses and children [3], and that hypothyroidism alters synaptic development and function [31-35]. TH insufficiency is known to lead to learning and memory deficits [5]. Our group has shown in adult rats that developmental iodine deficiency and hypothyroidism impairs LTP in the CA1 region [32]. However, the underlying mechanisms are still unknown. Considering that doublecortin regulates the migration of cortical neurons via actions at the distal ends of neurites that promote neurite extension [36,37], it is conceivable that doublecortin may be involved in neural developmental deficits caused by iodine deficiency and hypothyroidism. Similarly, since NCAM is critical in regulating morphogenetic processes during CNS development including neuronal migration and layering, and axonal growth, guidance and fasciculation [38], we hypothesize that NCAM-180 may be implicated in the brain damage induced by iodine deficiency and hypothyroidism. To this end, the present study has explored the changes of doublecortin and NCAM-180 expressions in hippocampus following developmental iodine deficiency and PTU-induced hypothyroidism. Our data show that developmental iodine-deficiency and hypothyroidism cause irreversible mal-regulation of doublecortin and NCAM-180 a few weeks before the obvious injury of nerve fibers in the hippocampus, implying that developmental iodine deficiency and hypothyroidism may impair the expression of doublecortin and NCAM-180, leading to disfunction of nerve fibers and hippocampal development.

## Methods

### Animals

Wistar rats (250-280 g) were obtained from the Center for Experimental Animals at China Medical University (Shenyang, China), with a National Animal Use License number of SCXK-LN 2003-0009. All experiments and surgical procedures were approved by the Institutional Animal Care and Use Committee at China Medical University, which complied with the National Institute of Health Guide for the Care and Use of Laboratory Animals. Rats were housed at an environmental temperature of 24 ± 1°C on a 12/12-h light/dark cycle and had access to food and water *ad libitum*. Animals were kept for 1 week before mating (♀:♂ = 2:1). The day when the vaginal plug was discovered was considered as gestational day (GD) 0. The pregnant rats were randomly assigned to four groups (n = 7 per dose group): control group, iodine-deficient group, 5 ppm PTU-treated group, or 15 ppm PTU-treated group. The control group was fed a normal diet (iodine content measured by As(3+)-Ce(4+) catalytic spectrophotometry: 470.50 ± 46.52 ng I/g food) and given tap water during the experiment. The iodine-deficient group was fed an iodine-deficient diet (as from an endemic area of severe iodine-deficient disease, iodine content: 14.11 ± 1.96 ng I/g food) and given tap water from GD6 until postnatal day (PN) 28. Rats of the PTU-treated groups were given 5 ppm or 15 ppm PTU (Sigma, St. Louis, MO, USA) in the drinking water and fed a normal diet from GD6 to PN28. The animal diet was comprised of corn (46%), rice (40%), soybean (13%), calcium carbonate (0.5%), and sodium chloride (0.5%). A normal diet and water was fed to all groups from PN28 to PN42.

Each litter was culled to 9-10 pups on PN4 (equal numbers of males and females in each group when possible). On PN14, PN21, PN28, or PN42, 8 pups in each group were weighted and anesthetized by etherization. Heart blood samples were obtained from 8 pups in each group at each time point, and serum was separated (3,000× g, 5 min) and stored at -70°C for subsequent measurement of TH with a supersensitive chemiluminescence immunoassay (IMMULITE; Diagnostic Products Corporation, Los Angeles, CA, USA).

### Histochemistry

On PN14, PN21, PN28, and PN42, the brains of 5 rats per group were preserved via intracardiac perfusion with 50-100 ml normal saline containing 0.02% heparin, followed by 200-400 ml 4% paraformaldehyde in 0.1 M potassium phosphate buffer (pH 7.4). Then, the brains were quickly removed from the skull and fixed overnight in the same fixative. The fixed brains were embedded in paraffin and sectioned into 6-μm-thick coronal sections. Brains were sectioned in a serial manner when intact structure of the hippocampus was observed in the slices. Every fifth/sixth slice was collected per animal on gelatin-coated microscope slide. Three sections of each rat brain were selected randomly for silver staining. After deparaffinization in xylene for 10 min followed by 100% ethanol, slides were washed in deionized water. The slides were incubated in 20% silver nitrate at 40°C for 40 min in the dark [39,40]. Concentrated ammonium hydroxide was added to the silver nitrate solution until the initial precipitate disappeared. The slides were incubated in this solution for 5 min in the dark, followed by incubation in a physical developer solution (10% potassium sodium tartrate) for 5 min. The slides were rinsed in gold chloride solution and fixed in 5% sodium thiosulfate solution. The sections were microscopically analyzed at 40× (objective 4× and ocular 10×) magnification for the hippocampal sub-regions of interest: CA1, CA3, and DG. The boundaries for the sub-regions were determined according to the Paxions-Wastson atlas of the rat brain [41]. Then images were obtained at a magnification of 400× (objective 40× and ocular 10×) to analyze the nerve fibers (stained black) and cell bodies.

### Western blotting

The brains of the 3 pups per group at each measured day were rapidly dissected from the skull and immediately submerged in an ice-cold artificial cerebrospinal fluid (aCSF: 124 mM NaCl, 3 mM KCl, 2 mM CaCl_2_, 1 mM MgSO_4_, 1.25 mM NaH_2_PO_4_, 26 mM NaHCO_3_, and 10 mM glucose, pH = 7.4). The hippocampus was removed and dissected on ice rapidly. With the help of a dissecting microscope, the hippocampus was bisected and the dorsomedial half was divided into four slabs cut perpendicular to the long axis of the hippocampus. According to the Paxions-Wastson atlas of the rat brain [41], each slab was dissected into CA1, CA3 and DG regions [42,43]. A tissue sample of each rat was homogenized in 250 μl of buffered isotonic cocktail containing protease and phosphatase inhibitors (50 mM Tris-HCl, 150 mM NaCl, 1% NP40, 0.1% SDS, 0.5% sodium deoxycholate, 10 mM NaF, 1 mM EGTA, 1 mM EDTA, and 0.2 mM PMSF). The sample was sonicated and incubated on ice for 30 min and then centrifuged at 13,000× g for 10 min at 4°C. The resulting supernatant was re-centrifuged and saved. The protein was estimated by Coomassie brilliant blue staining [44]. Samples were stored at -70°C until analyzed.

Tissue lysates of each rat sample were diluted to the protein concentration of 3 μg/μl and were boiled for 5 min. Ten μl aliquots of each sample (30 μg protein total) were loaded onto 10% SDS-acrylamide gels. Proteins were separated by application of a constant voltage of 100 V for 1.5 h and then transferred onto nitrocellulose membranes at a constant voltage of 10 V for 45 min. After blocking the nonspecific sites with PBS containing 0.1% Tween 20 (PBST) and 5% defatted dried milk, membranes were washed and incubated with primary antibody (rabbit anti-doublecortin, 1:1000 dilution, polyclonal, Cell Signaling Technology, Danvers, MA, USA; rabbit anti-NCAM, 1:1000 dilution, polyclonal, Millipore, Billerica, MA, USA; rabbit anti-β-actin, 1:1000 dilution, polyclonal, Santa Cruz Biotechnology, Santa Cruz, CA, USA) for 2 h at room temperature, then incubated with horseradish peroxidase (HRP)-conjugated secondary antibody (goat anti-rabbit, 1:5000 dilution, Zhongshan Biotechnology, Beijing, China). Blots were developed with the Easy Enhanced Chemiluminescence (ECL) Western Blot Kit (Transgen Biotech, Beijing, China). Initial control experiments determined the optimal time for exposing the blot to film, which was maintained throughout the experimental procedure. Membranes were exposed to film for the optimal time for each antibody and developed. Protein bands were subsequently quantified with an image analysis program (Gel Image System Ver. 4.00) and the data were recorded, with the net optical density corrected for background chemiluminescence. For each blot, the β-actin lanes were analyzed as a quality control. The signals from target bands on each blot were normalized to the mean signal of the quality control sample bands in order to simplify the comparisons across the blots and reduce inter-gel variability.

### Statistical analysis

All the statistical analyses were performed using SPSS software (Version 12.0; SPSS Inc, Chicago, IL) with the analyzer blind to the treatment of each group. Data were expressed as mean ± standard deviation (SD) and considered statistically significant at p < 0.05. To verify consistent protein loading among the gels, some blots were normalized against the β-actin expression and then the ratio with doublecortin or NCAM-180 was determined. There was no significant statistical difference between density percentage of the control and the ratio of β-actin; therefore, the analyses for the percentage of control are presented for each blot. At each time point, comparisons of pups' body weights, TH levels, and protein levels of doublecortin and NCAM-180 were made using one-way ANOVA. When the F-value indicated significance, least-significant difference (LSD) post hoc comparisons were made as appropriate to correct for multiple comparisons. All p-values were two-tailed.

## Results

### Animal models

Many studies have shown that PTU treatment reduces offspring body weight [35,45]. In line with these studies, our present data demonstrated that the offspring's body weights of the iodine-deficient and PTU-treated pups were lower than those of controls from PN14 to PN42, with a reduction of 18%-76% [43]. Reductions in TH were also observed in hypothyroid offspring [35]. From PN14 to PN28, the iodine-deficient, 5 ppm and 15 ppm PTU-treated pups had significantly lower TH levels than the controls, with a decrease of 49%-71%, 11%-67%, and 44%-73%, respectively [43]. On PN42, after diets were again normal, the serum TH concentrations returned to normal in all iodine-deficient and PTU-treated groups [43]. Taken together, in our hands, the iodine-deficient and PTU-treated diets caused pup hypothyroidism with developmental delay, which is consistent with the literature [43].

### Iodine deficiency and hypothyroidism damaged hippocampal nerve fibers

**Figure 1:**
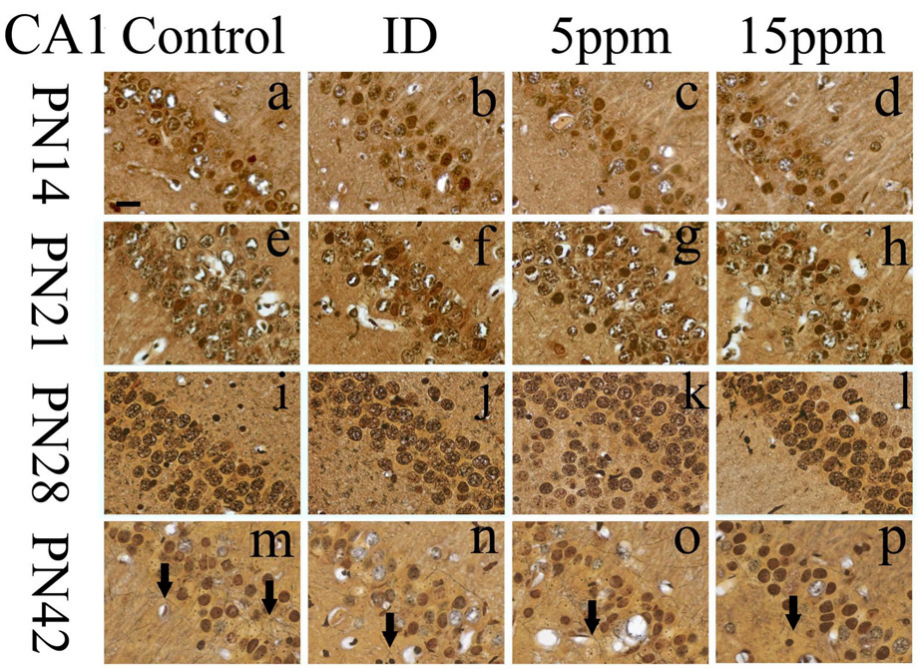
**Iodine deficiency and hypothyroidism damaged nerve fibers in CA1 region**. Silver staining was used to assess the damages of nerve fibers in CA1 region on PN14, PN21, PN28, and PN42 (n = 5). ID: Iodine-deficient group. On PN14, PN21, and PN28, cell bodies were arranged in order in each group, and fewer nerve fibers were observed. No markedly differences were observed between the control and the treatment groups. On PN42, nerve fibers (labeled with arrows) degeneration became apparent between the groups. Nerve fibers in iodine-deficient (Figure 1n), 5 ppm PTU-treated (Figure 1o), and 15 ppm PTU-treated (Figure 1p) groups were arranged in disorder, distorted, or disrupted, while nerve fibers in the controls were arranged in order with no obvious pathological changes (Figure 1m). Meanwhile, more nerve fibers were observed in the controls than the treatment groups. Scale bar represents 10 μm at lower left in (a) and applies to all panels.

**Figure 2:**
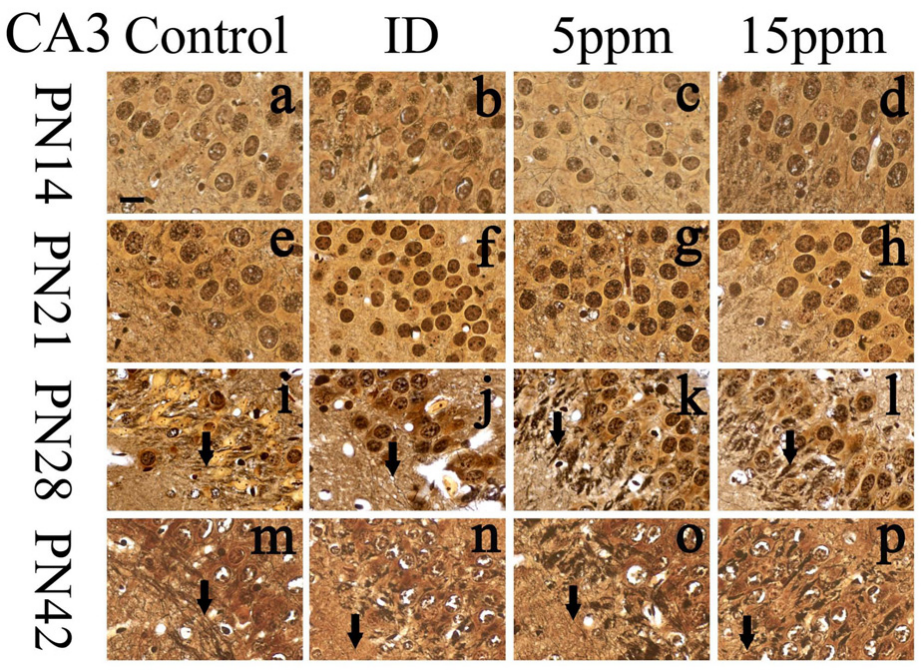
**Iodine deficiency and hypothyroidism impaired nerve fibers in CA3 region**. ID: Iodine-deficient group. On PN14 and PN21, cell bodies were arranged in order in each group, and fewer nerve fibers were observed. No markedly differences were observed between the control and the treatment groups (n = 5). On PN28 and PN42, nerve fibers (labeled with arrows) degeneration was obvious to identify between the groups. In iodine-deficient (Figure 2j&2n), 5 ppm PTU-treated (Figure 2k&2o), and 15 ppm PTU-treated (Figure 2l&2p) groups, nerve fibers were arranged in disorder, distorted, or disrupted, and some of them were concentrated and fused, while in the controls (Figure 2i&2m) were arranged in order and distinct. Scale bar represents 10 μm at lower left in (a) and applies to all panels.

**Figure 3:**
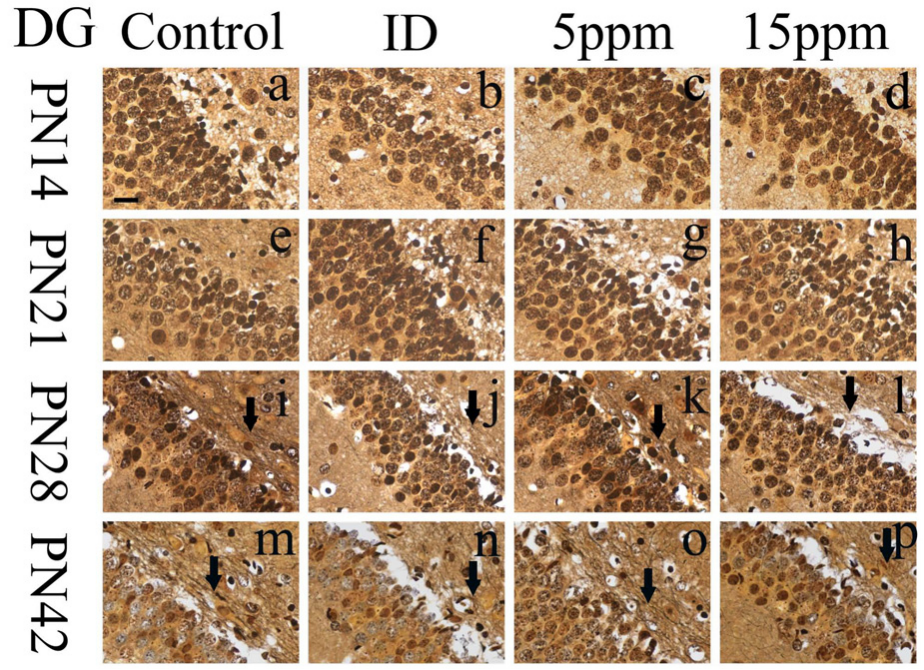
**Iodine deficiency and hypothyroidism damaged nerve fibers in DG region**. ID: Iodine-deficient group. On PN14 and PN21, cell bodies were arranged in order in each group, and fewer nerve fibers were observed. No markedly differences were observed between the control and the treatment groups (n = 5). On PN28 and PN42, silver-stained fibers (labeled with arrows) degeneration became apparent. Nerve fibers in iodine-deficient (Figure 3j&3n), 5 ppm PTU-treated (Figure 3k&3o), and 15 ppm PTU-treated (Figure 3l&3p) groups were arranged in disorder or disrupted, while nerve fibers in the controls (Figure 3i&3m) were arranged in order and distinct. Fewer nerve fibers were observed in the treatment groups than the controls. Scale bar represents 10 μm at lower left in (a) and applies to all panels.

Neuronal degeneration is characterized by increased sensitivity to silver staining [46]. To explore the effects of iodine-deficiency and hypothyroidism on hippocampal neurons, we analyzed the silver-stained neurons in the CA1 (Figure 1), CA3 (Figure 2), and DG (Figure 3) regions. On PN14 and PN21 in all groups, there was no sign of pathological lesions, where cell bodies were easily observed and arranged in good order. However, nerve fibers were much fewer in number in treated groups.

On PN28, compared with the control group, nerve fibers were found injured in the treated groups of regions CA3 and DG, as well as reduced in number (Figures 2j&2k&2l&3j&3k&3l). Injury appeared as fibers broken, disordered, bundled together, or fused with other fibers. In the control group, healthy nerve fibers were observed in all regions (Figures 2i&3i). The control nerve fibers were arranged in a good order with clear processes. Unlike regions CA3 and DG, nerve fibers in region CA1 were not yet damaged on PN28.

By PN42, all regions of treated pups, CA1, CA3, and DG, contained damaged nerve fibers (Figures 1n&1o&1p&2n&2o&2p&3n&3o&3p). Control groups of all regions of PN42 remained normal (Figures 1m&2m&3m). Our data demonstrate that developmental iodine deficiency and hypothyroidism can cause morphological damage in the hippocampus (CA1) even after a normal diet is restored (PN28 through PN42).

### Iodine deficiency and hypothyroidism down-regulated doublecortin

**Figure 4:**
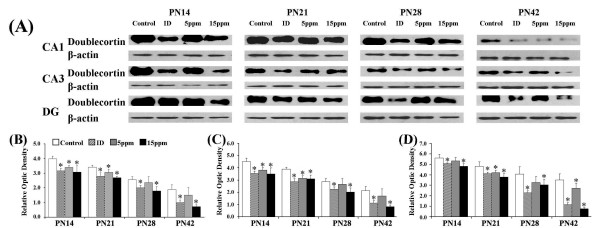
**Iodine deficiency and hypothyroidism down-regulated doublecortin in hippocampal subregions**. ID, iodine-deficient group. The upper bands (A) depict representative findings for rats subjected to developmental iodine deficiency and PTU-induced hypothyroidisms. The lower bar graphs show the results of the semi-quantitative measurement of doublecortin following developmental iodine deficiency and PTU-induced hypothyroidisms, for the CA1 (B), CA3 (C), and DG (D) regions. The height of each bar represents the mean ± SD for the groups. Within each time point, * indicates a significant difference from the control group, p < 0.05 (n = 3).

Doublecortin is a required component of migrating neurons which is accordingly highly expressed during foetal and neonatal stages of brain development [21]. Therefore, we chose doublecortin, a biomarker for neuronal development, to investigate how iodine deficiency and hypothyroidism affect neural differentiation. On PN14, PN21, PN28, and PN42, doublecortin was expressed in the CA1, CA3, and DG regions of the hippocampus (Figure 4A). Western blotting revealed a single reactive band with an approximate molecular weight of 45 kDa, as anticipated. On PN14, PN21, PN28, and PN42, a significant downregulation of doublecortin was observed in rats exposed to iodine-deficient and 15 ppm PTU-treated groups in CA1 (Figure 4B; PN14: F(3,8) = 7.62, p < 0.05; PN21: F(3,8) = 10.36, p < 0.01; PN28: F(3,8) = 4.40, p < 0.05; PN42: F(3,8) = 7.92, p < 0.01), CA3 (Figure 4C; PN14: F(3,8) = 6.62, p < 0.05; PN21: F(3,8) = 11.47, p < 0.01; PN28: F(3,8) = 4.32, p < 0.05; PN42: F(3,8) = 7.92, p < 0.01), and DG (Figure 4D; PN14: F(3,8) = 4.46, p < 0.05; PN21: F(3,8) = 6.35, p < 0.05; PN28: F(3,8) = 6.00, p < 0.05; PN42: F(3,8) = 32.58, p < 0.01) regions.

### Iodine deficiency and hypothyroidism up-regulated NCAM-180

**Figure 5:**
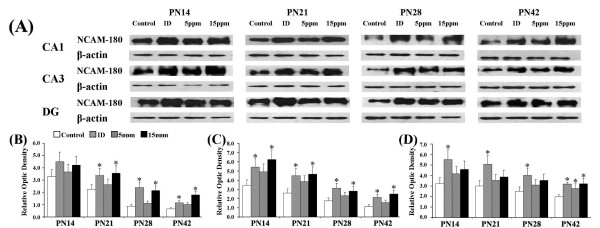
**Iodine deficiency and hypothyroidism up-regulated NCAM-180 in hippocampal subregions**. ID, iodine-deficient group. The upper bands (A) are representative findings for pups following developmental iodine deficiency and hypothyroidisms. The lower bar graphs indicate the results of the semi-quantitative measurement of NCAM-180 induced by developmental iodine deficiency and hypothyroidisms, for the CA1 (B), CA3 (C), and DG (D) regions. The height of each bar represents the mean ± SD. At each time point, * indicates a significant difference from the control group, p < 0.05 (n = 3).

NCAM-180, one of NCAM isoforms, has been shown to play an important role in synaptic remodeling accompanying LTP [27]. On PN14, PN21, PN28, and PN42, NCAM-180 was expressed in the CA1, CA3, and DG regions (Figure 5A). On PN14, PN21, PN28, or PN42, a significant upregulation of NCAM-180 were observed in rats exposed to iodine-deficient and/or PTU-treated groups in CA1 (Figure 5B; PN14: F(3,8) = 1.97, p > 0.05; PN21: F(3,8) = 4.38, p < 0.05; PN28: F(3,8) = 19.14, p < 0.01; PN42: F(3,8) = 15.67, p < 0.01), CA3 (Figure 5C; PN14: F(3,8) = 5.48, p < 0.05; PN21: F(3,8) = 5.62, p < 0.05; PN28: F(3,8) = 5.67, p < 0.05; PN42: F(3,8) = 10.14, p < 0.01), and DG (Figure 5D; PN14: F(3,8) = 4.67, p < 0.05; PN21: F(3,8) = 5.13, p < 0.05; PN28: F(3,8) = 4.12, p < 0.05; PN42: F(3,8) = 6.60, p < 0.01) regions.

## Discussion

The major findings of this study with rats exposed to developmental iodine deficiency or PTU-induced hypothyroidism were that (1) nerve fibers in the hippocampal CA1, CA3, and DG regions were impaired, (2) the levels of doublecortin were lower than controls, and (3) the levels of NCAM-180 were increased. We have previously shown that the concentrations of serum TH in the iodine-deficient and PTU-treated groups gradually returned to control levels by PN42 [43]. However, the recovery of TH on PN42 did not rescue the alterations in doublecortin and NCAM-180 in the hippocampus regions. These findings are in consistent with the irreversible hippocampal injuries following developmental iodine deficiency and PTU-induced hypothyroidism in rats [47,48].

Iodine deficiency is the most frequent cause of preventable brain damage in the world. However, remedies to eliminate iodine deficiency disorders are slow to develop [3]. Fetuses and infants among the population at high risk for iodine deficiency and its associated reduced intellectual ability [49]. It is generally accepted that the rapid rate of brain growth in the late gestation and early postnatal stages make the immature brain susceptible to iodine and subsequent thyroid deficiency [49]. Our previous study has shown that iodine deficiency is still a serious public health problem in China [50]. The present study is a follow-up project to explore neuronal degeneration in two rat models following developmental exposure to low levels of TH.

TH is essential for brain differentiation processes, including dendritic and axonal growth, synaptogenesis, neuronal migration, myelination, and expression of specific mature neuron proteins [51]. Inadequate supply of TH to the developing brain leads to several histological changes in the brain, such as decreased axonal density, reduced numbers of dendrites and dendritic spines, and delayed synaptogenesis [52,53]. Our previous study revealed that low circulating TH levels induce deleterious hippocampal morphology changes in the CA1, CA3, and DG regions on study days PN21, PN28, and PN42 [43]. In the current study, observation of the silver-stained neurons of the CA1, CA3, and DG regions showed that nerve fiber injuries were observed at later stages of brain development. In the silver-staining tissue sections there were far fewer healthy nerve fibers in the treatment groups compared to controls. It deserves noting that there was no visible nerve fiber injury in the early stages on PN14 and PN21. Currently the authors do not have evidence to explain this time delay. It could be due to the fact that most of the brain structures examined in these rats are formed by the third postnatal week [54]. Indeed, a delayed damage was also observed in the CA1 following transient ischemia [55]. Taken together with our previous analysis [43], we speculate that iodine deficiency and hypothyroidism may lead to deficits in neural development, degeneration of hippocampal nerve fibers, and thus an impairment of neuronal function.

It is clear that hypothyroidism results in stunted growth and impairs brain development [56,57]. A recent study has concluded that the hypothyroidism-induced developmental damages of the hippocampus take place in CA1, CA3, and DG regions [58]. In the hippocampus, DG granule cells project to the dendrites of pyramidal cells of CA3 via mossy fibers. At the same time, these cells contribute a major input to CA1 (the Schaffer collaterals). This circuit is implicated in different functions of hippocampus [58]. The neuronal progenitor cells in the dentate subgranular zone (SGZ) can differentiate into neurons and glial cells [59,60]. Some of the newborn neurons migrate to the granular layer and extend mossy fiber axons, indicating that they are integrated into the functional circuitry of the hippocampus [61-64]. Given that doublecortin is a marker of new neurons and expresses in differentiating and migrating neurons [17], a reduction of doublecortin by dietary iodine deficiency and PTU-induced hypothyroidism in the present study is in agreement with a reduction in nerve fibers and newborn neurons. Our data suggest a connection between hippocampal morphology injuries and iodine deficiency and hypothyroidism. Considering that a reduction of newly born granule cells may influence mossy fiber innervations in the CA3 region, it is conceivable that iodine deficiency and hypothyroidism may subsequently impair neuronal connectivity, pyramidal cell excitability and memory formation [65-67] by reducing newborn granule cells and nerve fiber innervations in the hippocampus.

NCAM is a cell adhesion molecule that mediates homophilic adhesion between cells to regulate CNS development [22,23]. The NCAM isoform, NCAM-180, plays an important role in synaptic remodeling and LTP [27]. Hence, NCAM plays an important role in structural remodeling of the nervous system, and one of these important functions is to hold developing neurites together during neuron outgrowth and formation of neural connections [68]. It has been shown that both doublecortin and NCAM are regulated by TH via a classical genomic molecular mechanism [30]. Indeed, this finding is in agreement with our observations of the implications of the two proteins in the structural injuries in the hippocampus following developmental iodine deficiency and hypothyroidism in rat pups. In addition, our data of upregulation of NCAM expression by iodine deficiency and hypothyroidism is in consistent with the literature where hypothyroidism disrupts cell migration [69,70]. As we know that cell adhesion molecules serve as guidance clues for migration and axonal growth [30], via which the migrating neurons interact with extracellular matrix proteins to find the migration path. Therefore, NCAM can hold developing neurites together during neuron outgrowth and formation of neural connections [68]. So, hypothyroidism-induced NCAM-upregulation may alter the normal neuronal development, including the nerve fiber injuries. However, so far, the authors do not know the mechanisms underlying hypothyroidism-upregulated NCAM expression in the hippocampus. It could be due to NCAM gene upregulation by hypothyroidism [30]. There is also another piece of evidence supporting the transcriptional upregulation of NCAM in the neonatal rat brain by hypothyroidism [38]. Furthermore, it cannot be excluded that iodine deficiency and PTU-induced hypothyroidism may fail to suppress the activation of NCAM-180, leading to the up-regulation of NCAM-180 expression [30].

Concerning NCAM expression, we did see a discrepancy with the literature, where Gilbert and colleagues have shown that PTU starts to upregulate NCAM 1 genes at 3 ppm [30]. In contrast, in the present study NCAM-180 protein expression is not sensitive to PTU at 5 ppm. The authors do not have an answer for this difference, but it could be due to protein expression changes being less sensitive than the gene regulations. In addition, it should also be taken in account that this paper used a different test method, sampled at a different time point, and investigated different hippocampus regions from the Gilbert study [30].

Interestingly, we observed doublecortin decrease and NCAM-180 increase on PN42 when TH levels were restored to a normal state, suggesting irreversible hippocampus impairment. This is in line with many pieces of evidence, where maternal iodine deficiency during pregnancy and lactation are confirmed as one of the causes of irreversible CNS damage in offspring [43,45,47,48,71,72]. In summary, we used two rat models to show that developmental iodine-deficiency and hypothyroidism causes irreversible mal-regulation of doublecortin and NCAM-180 a few weeks before the obvious injury of nerve fibers in the hippocampus, demonstrating that the developmental iodine deficiency and hypothyroidism impair the expression of doublecortin and NCAM-180, leading to nerve fiber malfunction and thus the impairment in hippocampal development. A detailed mechanism is still lacking by which developmental iodine deficiency and hypothyroidism regulate doublecortin and NCAM proteins and deserves further investigation.

## Conclusion

Developmental iodine deficiency and hypothyroidism depress the expression of doublecortin and enhance the NCAM-180, leading to nerve fiber morphology abnormalities and probable malfunction, and thus impairments in hippocampal development.

## Abbreviations

aCSF: artificial cerebrospinal fluid; ANOVA: analysis of variance; CNS: central nervous system; DG: dentate gyrus; ECL: Easy Enhanced Chemiluminescence; FT3: free triiodothyronine; FT4: free thyroxine; GD: gestational day; HRP: horseradish peroxidase; LSD: least-significant difference; LTP: long-term potentiation; NCAM: neural cell adhesion molecule; NCAM-180: neural cell adhesion molecule-180; PBS: phosphate-buffered saline; PBST: PBS containing 0.1% Tween 20; PN: postnatal day; PTU: propylthiouracil; RXR: retinoic acid X receptor; SD: standard deviation; SGZ: subgranular zone; T3: triiodothyronine; T4: thyroxine; TH: thyroid hormones; TR: thyroid hormone receptor.

## Competing interests

The authors declare that they have no competing interests.

## Authors' contributions

JG, WL, and JC conceived of the study, and participated in its design and coordination. JG drafted the manuscript. JG, JD, YW, HX, WW, and JZ carried out the experiments, collected and analyzed the data. JC and QX revised the manuscript. All authors participated in writing, and read and approved the final manuscript.
